# The Synergistic Effects of Organizational Justice and Trust to Supervisor on Vagal Tone: Preliminary Findings of an Empirical Investigation

**DOI:** 10.3390/ijerph16050790

**Published:** 2019-03-04

**Authors:** Raphael M. Herr, Jian Li, Peter Angerer

**Affiliations:** 1 Mannheim Institute of Public Health, Social and Preventive Medicine, Medical Faculty Mannheim, Heidelberg University, 68167 Mannheim, Germany; 2 Institute of Occupational, Social and Environmental Medicine, Centre for Health and Society, Faculty of Medicine, Heinrich-Heine-University of Düsseldorf, 40225 Düsseldorf, Germany; Jian.Li@uni-duesseldorf.de (J.L.); Peter.Angerer@uni-duesseldorf.de (P.A.); 3 Department of Environmental Health Sciences, Fielding School of Public Health; School of Nursing, University of California Los Angeles, Los Angeles, CA 90095, USA

**Keywords:** organizational justice, vagal tone, heart rate variability, trust to supervisor, synergistic effects

## Abstract

The influence of perceived unfairness at the workplace (organizational injustice) on employee health is well established. Several theories explain the unpleasant and stressful nature of the experience of injustice, using trust as a central element. This study examines the effect of trust to supervisor on the association of perceived injustice with vagal tone—an objective marker for stress experience. Questionnaires assessed organizational justice and trust. Vagal tone was measured by indictors of heart rate variability (HRV), which captured parasympathetic (pNN50, RMSSD, and HF) and parasympathetic and sympathetic (SDNN, and LF) regulation. Synergistic effects were tested by linear regressions with interaction terms between organizational justice and trust to supervisor in 38 managers. Organizational justice was related to HRV indicators that reflect in particular the parasympathetic branch (β_pNN50_ = 0.32, *p* < 0.05; β_RMSSD_ = 0.27, *p* < 0.1), and interaction effects with trust to supervisor were also most pronounced there (interaction β_pNN50_ = −0.41, *p* < 0.01; β_RMSSD_ = −0.47, *p* < 0.01). In conclusion, the combination of low perceived justice and trust to supervisor appears substantial to the physiological stress threat of employees. Promoting fairness at the workplace might reduce stress; if not possible, trust to supervisor should be enhanced.

## 1. Introduction

The perception of unfairness at the workplace (organizational injustice) has consistently been linked to various job-related factors, such as commitment, turn-over intention, motivation and productivity, as well as to adverse mental and physical health [1,2,3,4]. The negative health consequences are seen to be the result of the negative emotions and stress associated with the perception of injustice [3,5]. 

Several theoretical concepts have been developed to explain why people are interested in fairness and why their perception of unfairness might be stressful to them. The fairness heuristic theory argues, for example, that perceived justice at work is important to employees because of facing the “fundamental social dilemma”, which is expressed in the question of whether to trust and cooperate with authorities [6]. Therefore, “perceptions of fairness will be used as a shortcut to deciding whether to accept the authority’s decision or reject it” [7]. Accordingly, justice can be seen as an employee’s proxy for trust to supervisor and a prerequisite for the willingness to cooperate. In this context, fairness is used as a heuristic because the trustworthiness of an authority is difficult to judge since it depends on not directly observable concepts, such as integrity, benevolence, and ability [8]. In consequence, the absence of fairness signals a lack of trustworthiness of an authority, accompanied by higher levels of uncertainty. Related to this reasoning and building upon the fairness heuristic theory, the uncertainty management theory argues fairness to be a heuristic to deal with uncertainty at work in general [9]. Justice and subsequent trust thus help employees to manage general work uncertainty [10]. The concept of trust is also central to the leading theoretical framework for the importance and health effects of organizational justice: the social exchange theory [11,12,13]. According to this theory, social relationships are understood as long-term exchanges of resources (or favors) with a diffuse obligation to reciprocate [14,15]. Perceived organizational fairness is seen as a prerequisite for such a social exchange, while trust is an essential part because it can reduce uncertainty about the others’ reciprocity and obligations [10,14,16]. 

All these theories establish a close link between justice and trust, a link, which is also empirically confirmed in the field of organizational psychology [1,2,17]. Increasing evidence indicates that trust transmits the effects of justice [10,14,18]. In this context, trust might have the potential to reduce the uncertainty of injustice, which enhances predictability and lowers levels of distress [10,16,19,20].

The physiological manifestation of prolonged high stress levels can be indexed by vagal tone. Persistent stress is accompanied by a dysregulation of the autonomous nervous system (ANS) in terms of a shift of the relation of its two branches: the sympathetic nervous system (SNS) and parasympathetic nervous system (PNS). The SNS ensures maximum performance, for example, by increasing heart rates and blood glucose levels. The PNS, by contrast, provides relaxation and recovery through reduced heart rates. Chronic stress can lead to chronic over-activation of the SNS (the parasympathetic is not sufficiently employed), leading to cardiac disease, including myocardial infarction, cardiac sudden death, and cardiovascular disease (CVD) (e.g., [21,22,23,24]). Dysregulation of the ANS can be indexed by heart rate variability (HRV) through indicating PNS activity, also called vagal tone [25]. Evidence shows HRV to be related to adverse psychosocial working conditions, including perceived organizational injustice [16,26,27].

Based on the reasoning above, this empirically psychophysiological study examined the hypothesis that associating organizational justice with vagal tone, as indexed by HRV, depends on the degree of trust to supervisor in difficult situations. We hypothesize that HRV is lowest if perceived justice and trust to supervisor are low, while high trust (or justice) can compensate for low justice (or trust) reflected in HRV levels comparable to the condition in which both are high. 

## 2. Materials and Methods 

### 2.1. Participants

This study uses data of a cohort followed up after a preceding stress management intervention. Employees of a manufacturing plant in Southern Germany were invited in July 2006 to participate in a stress management training program. This program was designed to improve the ability to identify and cope with workplace stressors [28]. Participants were followed up after this training in three waves: 2008, 2015, and 2016. In the last wave in 2016 organizational justice, trust to supervisor, and the heart rate (HR) were measured. Of the 63 persons participating in this wave, 49 persons volunteered HR measurements. After excluding persons with missing or invalid data on questionnaire scales (organizational justice, trust to supervisor (*n* = 3)) and HRV measurements (artifact ratio > 5% (*n* = 5) and measurement quality < 50% (*n* = 2)) and the only female, 38 male middle managers remained for analyses. All participants provided written informed consent, and the ethical committee of the Heinrich-Heine-University of Düsseldorf approved the study (no. 5684).

### 2.2. Methods

Organizational justice was measured by the validated German organizational justice questionnaire (G-OJQ) [29]. This 11-item scale (seven items capture the procedural justice dimension and four items are related to the interactional justice one) asked participants to indicate on a 5-point Likert scale to what extent each statement applies to their work situations (ranging from ‘‘does not apply at all’’ to ‘‘applies completely’’). Example items are “The supervisor makes decisions that are free of personal biases” or “Everyone has the opportunity to question decisions that are made”. A mean score was calculated with lower values indicating lower organizational justice perceptions and higher values denoting higher organizational justice perceptions (Cronbachs’ α = 0.90).

The trust to supervisor was assessed by one item taken from the Short Questionnaire for Work Analysis [30]. The item asks: “I can trust my direct supervisor when things get difficult at work” (1 = does not apply at all, 2 = does rather not apply, 3 = partially applies, 4 = largely applies, 5 = fully applies). 

The HR was measured by Faros devices, which were attached by means of chest strap to the participants at the same day they received the questionnaire. On the next day, participants returned the device. HRV indices were calculated by relevant software, which has been applied to recent research [31,32,33]. An overview of the indices used is presented in Table 1. Three indices measure HRV in the time domain (pNN50, RMSSD, and SDNN) and two in the frequency domain (LH and HF); pNN50, RMSSD, and HF primarily reflect the PNS, while SDNN and LF indices reflect the SNS and PNS.

As potential confounders age (in years), job position, height and weight (to calculate body mass index (BMI)), and smoking behavior (yes/no) were assessed by questionnaire. Job position was classified as segment leader vs. other positions.

### 2.3. Statistical Analyses

To approach normal distributions, HRV indicators were logarithmically transformed, outliers (standard deviations of ±3.5) were removed, and variables were z-transformed for analyses. Linear regression models estimated associations of organizational justice with HRV indices in three sequential steps. The first step estimated the association of organizational justice with the different HRV indices, while the second step also included trust to supervisor. In the last step, a multiplicative interaction term between organizational justice and trust to supervisor was included to assess synergistic effects. Two models of adjustment with potential a priori defined confounders were calculated [26]. The first model (Model 1) controlled for age, while the second model (Model 2) additionally adjusted for job position, smoking behavior, and BMI. Analyses were performed using SPSS 25 (IBM Corp. Released 2017. IBM SPSS Statistics for Macintosh, Version 25.0. Armonk, NY, USA).

## 3. Results

Characteristics of the study population and mean values for organizational justice, trust to supervisor, and the HRV indices are displayed in Table 2. 

**Figure 1:**
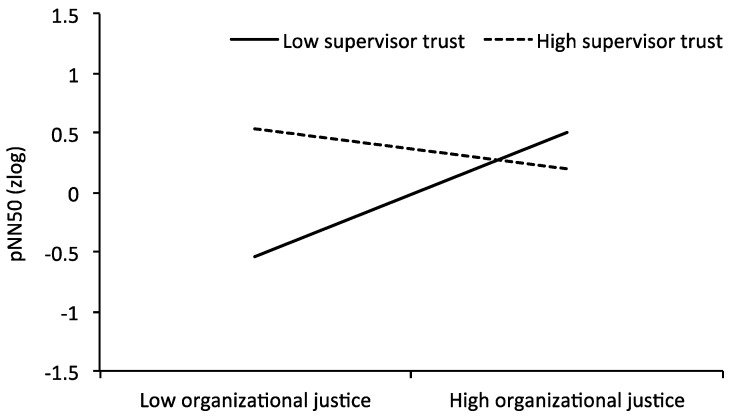
Synergistic effects of organizational justice and trust to supervisor on HRV (pNN50; adjusted for age (Model 1)).

In linear regression models (Table 3), a significant interaction term between organizational justice and trust to supervisor was observed for pNN50, RMSSD, and HF power, which were also independent of level of adjustment (all betas ≥ −0.37; *p*-values ≤ 0.05). As indicated in Figure 1, HRV was lowest in the case of low perceived justice and low trust to supervisor. In all other conditions, HRV indices were higher on a comparable level. These findings confirm our hypothesis.

## 4. Discussion

Our study attempted to provide empirical evidence examining the synergistic effects of low perceived organizational justice and trust to supervisor on reduced vagal regulation. We found that HRV was lowest (indicating most stress-related vagal dysregulation) when both organizational justice and trust to supervisor were low. HRV indices were on a higher and comparable level when organizational justice and/or trust to supervisor were high. This indicates that trust to supervisor can buffer the negative stress effects of low organizational justice. 

Several studies have established the influence of trust on the association of organizational justice with organizational outcomes, such as job satisfaction, turnover intentions, commitment, task performance, and citizenship behavior [14,34,35]. This study extends the literature to include stress-related physiological regulation: lowest HRV values were found when perceived justice and trust to supervisor were low. With fairness heuristic theory and uncertainty management theory as our basis, we have argued that perceived justice might be used as a shortcut for trust to supervisor, which appears hard to determine. The findings of our study suggest that increased trust to supervisor might buffer stress levels which result from low organizational justice. On the other hand, if persons rate their supervisor to be less trustworthy, high organizational justice might also compensate for this experience and reduce the stress load.

The reciprocal compensation of low justice and trust to reduce employee stress levels has relevant practical implications. Organizational justice perceptions might be improved by occupational interventions. For this purpose, Greenberg [36] identified three essential aspects. The first aspect refers to a dignified and respectful explanation to employees of the resource allocation in the company, while the second aspect states that employees should be given a voice and this voice should also be heard. The third aspect relates to the procedures in the company, which should be accurate, unbiased, and implemented transparently. However, occupational interventions to improve organizational justice face several challenges. Mangers are mostly unaware of injustice as a problem and tend not to address this topic [37]. Moreover, the acceptance and trust of the company, as well as the willingness to learn and change, are often lacking. These factors are essential both to change practices and to implement procedural justice rules (i.e., procedures should be consistent, without bias, accurate, correctable, representative, and ethical) to assure fairness of the decision-making process and a fair allocation of outcomes [37,38]. If it is not possible to implement such large-scale changes within a company, a remedy to buffer stress threat might be the improvement of trust to supervisor. 

Trust to supervisor and organizational justice were related to time-domain HRV indices reflecting PNS activation in particular (i.e., pNN50, and RMSSD). Regarding the frequency domain, an association with LF power, which reflects PNS and SNS activity, was also observed. However, significant interactions between justice and trust were found exclusively for PNS reflecting HRV indicators (pNN50, RMSSD, and HF power). This indicates that lacks of perceived organizational justice and of trust to supervisor might be related to reduced bodily recovery, potentially linking low perceived organizational justice to physical and mental ill-health [3,4,39]. 

The limited strength of associations between organizational justice, trust and HRV indices might raise the question about its clinical relevance. A recent dose–response meta-regression revealed that a 1% increase in HRV is associated with an approximately 1% lower risk of CVD [21]. Thus, even small changes in HRV indicators can have relevant effects and efforts to change the psychosocial work environment in terms of enhanced perceived justice and trust to supervisor have pertinence.

In this study, we admit that the assessment of trust was rather simplistic, which has been criticized in the past [40]. In the context of organizational justice, different types of trust appear relevant: trust in supervisor, trust in organizations, cognition-based trust (i.e., confidence in dependability, reliability, and professionalism) and affect-based trust (i.e., confidence in emotional investments, expressions of genuine care and concern, and an understanding of reciprocated sentiments) [10,14]. Further studies might consider how these (and potentially other) types of trust relate to vagal tone.

Several more limitations must also be reported. The cross-sectional design of the study prohibits causal inferences. The temporal reciprocal effects of justice and trust on stress load have to be disentangled in further longitudinal studies. In addition, due to the relatively small sample size restricted to men of a specific company in a specific position (sandwich position between higher management and production), this study must be considered as preliminary and further studies should include both women and other employee levels. Only one woman was available for our analyses; however, she was excluded because of known gender differences in justice perception (e.g., [41]). Another limitation refers to potential confounding factors, which were not included in analyses, like other CVD markers (such as blood pressure), or specific lifestyle factors (i.e., alcohol consumption and physical activity). Furthermore, HRV measures are only indirect assessments of autonomic activity. In addition, this cohort is a follow-up study of a preceding stress management intervention, potentially resulting in decreased injustice perceptions and a general improved health, leading to a selection bias. This might have restricted the measurement range and potentially led to an underestimation of the true associations. However, a comparison of the study sample with the persons who could not be followed up (attrition analyses) revealed no significant differences in key variables measured at baseline: demographics (age and education), professional variables (leadership responsibility, shift work, hours of overtime per month, daily break time, self-reported sick leave days), health status and behavior (BMI, intensive sports, and smoking), sleep quality, stress reactivity, effort–reward ratio, and mental health (anxiety and depression) (*p*-values > 0.10). The analytic sample and the drop-outs only differed significantly regarding the professional status (Χ^2^ = 9.7, *p* = 0.044); in the analytic sample were more segment leaders (51.4% vs. 32.1%) and less group leaders (2.7% vs. 23.4%). This overall suggests no strong selection bias. Lastly, multiple regression analyses might have inflated the Type I error rate. Bonferroni correction of the *p*-value for multiple testing (α/number of tests) [42] results in a *p*-value of 0.01 for an α of 0.05 and five HRV indicator tests. In consequence, especially the associations with *p*-values of ≤ 0.01 (i.e., two asterisks in Table 3) should be considered to be relevant.

## 5. Conclusions

In conclusion, low levels of perceived justice at the workplace combined with low trust to supervisor manifest in lowest vagal tone, indicating highest physiological stress load. The findings of our empirical investigation confirm theoretical considerations that fairness might be a heuristic for trust, and unfairness is especially stressful in the absence of trust to supervisor. Potential workplace interventions have two starting points to reduce the stress levels of employees: promoting organizational justice and/or enhancing trust to supervisor.

## Figures and Tables

**Table 1:** Denotation of applied heart rate variability (HRV) indices.

Domain	Abbreviation	Measurement Unit	Description	ANS Reflection
Time				
	pNN50	%	Percentage of successive RR intervals that deviate more than 50 ms	PNS
	RMSSD	ms	Root mean sum of squares of successive differences	PNS
	SDNN	ms	Standard deviation of all N-N intervals	SNS and PNS
Frequency				
	LF power	Hz	Low-frequency power	SNS and PNS, primarily SNS
	HF power	Hz	High-frequency power	PNS

ms = milliseconds; ANS = autonomic nervous system; SNS = sympathetic nervous system; PNS = parasympathetic nervous system; Hz = hertz.

**Table 2:** Characteristics of the study population.

Age, Years (Mean, SD)	40.66	6.44
Segment leader (*n*, %)	28.9%	11
Body mass index (mean, SD)	27.4	2.60
Non-smoker (mean, SD)	81.6%	31
Organizational justice (mean, SD)	3.69	0.64
Trust to supervisor (mean, SD)	4.11	0.83
pNN50 (mean, SD)	6.81	6.51
RMSSD (mean, SD)	26.41	9.98
SDNN (mean, SD)	147.31	34.25
HF power (mean, SD)	249.98	207.74
LF power (mean, SD)	1044.19	625.07

**Table 3:** Linear regression models for the association of organizational justice, trust to supervisor, and their multiplicative interaction with HRV indices.

Models	pNN50				RMSSD				SDNN				HF Power				LF Power			
	Model 1		Model 2		Model 1		Model 2		Model 1		Model 2		Model 1		Model 2		Model 1		Model 2	
	Beta	S.E.	Beta	S.E.	Beta	S.E.	Beta	S.E.	Beta	S.E.	Beta	S.E.	Beta	S.E.	Beta	S.E.	Beta	S.E.	Beta	S.E.
Step I																				
Organizational justice	0.320 *	0.154	0.251	0.156	0.277	0.159	0.209	0.161	0.06	0.169	0.036	0.18	0.259	0.16	0.19	0.164	0.295	0.155	0.214	0.149
Step II																				
Organizational justice	0.098	0.164	0.046	0.167	0.122	0.178	0.074	0.182	−0.102	0.19	−0.124	0.203	0.142	0.183	0.08	0.187	0.089	0.167	0.04	0.163
Trust to supervisor	0.453 **	0.166	0.430 *	0.174	0.316	0.181	0.283	0.19	0.33	0.193	0.335	0.212	0.239	0.186	0.23	0.195	0.418 *	0.17	0.364 *	0.17
Step III																				
Organizational justice	0.134	0.151	0.105	0.16	0.164	0.162	0.149	0.17	−0.084	0.19	-0.094	0.207	0.181	0.17	0.148	0.179	0.112	0.164	0.078	0.163
Trust to supervisor	0.215	0.177	0.205	0.193	0.043	0.19	0.002	0.206	0.214	0.223	0.221	0.251	−0.022	0.198	−0.026	0.217	0.268	0.192	0.221	0.197
Interaction justice × trust	−0.412 **	0.121	−0.374 *	0.134	−0.473 **	0.13	−0.468 *	0.143	−0.201	0.152	−0.190	0.173	−0.451 *	0.136	−0.426*	0.15	−0.260	0.131	−0.237	0.137

** *p* ≤ 0.01, * *p* ≤ 0.05. Model 1 adjusted for age. Model 2 adjusted for age, job position, BMI, and smoking behavior. S.E. = standard error.
